# Longitudinal Trajectories of Study Characteristics and Mental Health Before and During the COVID-19 Lockdown

**DOI:** 10.3389/fpsyg.2021.633533

**Published:** 2021-03-10

**Authors:** Llewellyn E. van Zyl, Sebastiaan Rothmann, Maria A. J. Zondervan-Zwijnenburg

**Affiliations:** ^1^Department of Industrial Engineering, University of Eindhoven, Eindhoven, Netherlands; ^2^Optentia Research Focus Area, North-West University, Vanderbijlpark, South Africa; ^3^Department of Human Resource Management, University of Twente, Enschede, Netherland; ^4^Institut für Psychologie, Goethe University, Frankfurt, Germany; ^5^Department of Methodology and Statistics, Utrecht University, Utrecht, Netherlands

**Keywords:** mental health, study resources, university students, COVID-19, piecewise latent growth modeling, coronavirus

## Abstract

The *COVID-19 lockdown* has significantly disrupted the higher education environment within the Netherlands and led to changes in available study-related resources and study demands of students. These changes in *study resources* and *study demands*, the uncertainty and confusion about educational activities, the developing fear and anxiety about the disease, and the implementation of the COVID-19 lockdown measures may have a significant impact on the *mental health* of students. As such, this study aimed to investigate the trajectory patterns, rate of change, and longitudinal associations between study resources–demands and mental health of 141 university students from the Netherlands before and during the COVID-19 lockdown. The present study employed a longitudinal design and a piecewise latent growth modeling strategy to investigate the changes in study resources and mental health over a 3 month period. The results showed that moderate levels of student resources significantly decreased before, followed by a substantial rate of increase during, lockdown. In contrast, study demands and mental health were reported to be moderate and stable throughout the study. Finally, the growth trajectories of study resources–demands and mental health were only associated before the lockdown procedures were implemented. Despite growing concerns relating to the negative psychological impact of COVID-19 on students, our study shows that the mental health during the initial COVID-19 lockdown remained relatively unchanged.

## Introduction

The COVID-19 pandemic has resulted in a global health crisis (Ebrahim et al., 2020). Officially declared a pandemic on the 11th of March 2020, it has resulted in more than 48 million confirmed infections and over 1.3 million deaths across 213 countries (World Health Organization (WHO), 2020). During the first wave of the pandemic, 380,000 people within the Netherlands were infected, 15,456 were admitted to hospitals, and 7,576 died due to COVID-19 [National Institute for Public Health and the Environment (RIVM), 2020b]; yet these statistics were said to underestimate the real impact and spread of the disease (Ebrahim et al., 2020). At the start of the pandemic, the Dutch Government’s strategy was primarily focused on containing the spread of the virus and protecting vulnerable groups (Antonides and van Leeuwen, 2020; Dutch Government, 2020; National Institute for Public Health and the Environment (RIVM), 2020a). With an ever-increasing case fatality rate (National Institute for Public Health and the Environment (National Institute for Public Health and the Environment (RIVM), 2020b), the COVID-19 has caused a severe public outcry and led to the implementation of unprecedented measures to prevent infection spread within the Netherlands (Dutch Government, 2020).

These measures ranged from social isolation, quarantine, local and international travel restrictions, border closures, large-scale event cancelations, and business, school, and university closures (Antonides and van Leeuwen, 2020; National Institute for Public Health and the Environment (RIVM), 2020b). This *COVID-19 lockdown* led to a significant disruption in all business sectors, where organizations were forced to close or radically change their *modus operandi* (de Haas et al., 2020). Dutch universities were not immune to these disruptions (Meeter et al., 2020). Universities restricted access to buildings, closed libraries, canceled lectures, delayed exams, and restricted access to virtual private networks required for assignments/exams (Maastricht University, 2020; Meeter et al., 2020). Moreover, universities were forced to adopt radically different forms of education (Dutch Government, 2020) where students needed to be taught via e-learning, examinations required online proctoring, contact with lecturers/peers were limited, assignments/examinations had to change, and students were bombarded with conflicting information from various sources (Meeter et al., 2020). These changes in students’ *study resources* (SR), the increased *study demands* (SD), the uncertainty and confusion about educational activities, development of fear caused by the ever-increasing COVID-19 infection/mortality rates, and the implementation of lockdown procedures may have a significant impact on their *mental health* (MH) (Fried, 2020; Roy et al., 2020).

Brooks et al. (2020) suggested that individuals who are kept in isolation/quarantine experience significant psychological distress, and it makes it difficult for individuals to engage in enjoyable social/work/leisure activities. Prolonged home confinement during the COVID-19 pandemic directly affects students’ physical health and MH through a reduction in physical activity, social contact, and exposure to daylight (Meeter et al., 2020; Tull et al., 2020; Wang et al., 2020). During the COVID-19 lockdown in China, people reported to spend more time in bed but paradoxically experienced lower sleep quality (Gao et al., 2020). Experiences of stress, depression, and anxiety also increased during the pandemic (González-Sanguino et al., 2020), which affected students’ motivation and the hours being spent on educational activities (Meeter et al., 2020). Further, social isolation increases loneliness, which directly affects cardiovascular functioning and leads to poor MH outcomes (Leigh-Hunt et al., 2017). To cope with social isolation and loneliness, individuals are drawn toward social media, but exposure has been shown to increases stress, depression, and anxiety during the COVID-19 outbreak in China (Gao et al., 2020). It is therefore not surprising that more than 80% of individuals reported MH problems/needs during the COVID-19 crisis; however, access to MH services have been severely restricted (Roy et al., 2020). Therefore, students may not have the necessary physical, psychological, or social resources needed to cope with the MH challenges associated with the COVID-19 pandemic (Jacobson et al., 2020).

Given the radical changes in SR and SD and the potential MH problems associated with the COVID-19 lockdown, it is imperative to understand how such develops over time. It is important to investigate how Dutch university students’ perceptions of SR/SD and MH changed before and during the COVID-19 lockdown.

## Literature Review

### MH of Students

Understanding the growth trajectory of MH during COVID-19 is imperative to ensure society’s optimal functioning (Zhang and Ma, 2020). MH, defined as “a syndrome of symptoms of positive feelings and positive functioning in life” (Keyes, 2002, p. 207), is an essential factor to consider when governments reflect upon intervention methods to manage/contain the impact of large-scale pandemics like COVID-19. Keyes et al. (2020) argued that MH is a function of *emotional* (i.e., life satisfaction and positive and negative affect), *psychological* (i.e., autonomy, environmental mastery, personal growth, positive relations with others, purpose in life, and self-acceptance), and *social well-being* (i.e., social acceptance, actualization, coherence, contribution, and integration), which refers to more than just the absence of psychopathology. Traditionally, MH was described as a single-lane continuum with psychopathology at the one end and well-being on the other end of the spectrum (Keyes et al., 2020). However, Seligman (2012) argued that the absence of a disease is not an adequate criterion to describe one as being either physically or psychologically healthy. Several studies have shown that individuals could report both high levels of psychopathology (e.g., narcissism) and high levels of life satisfaction (Greenspoon and Saklofske, 2001; Keyes, 2002; Seligman, 2012). Distress/pathology and well-being should therefore be seen as distinct yet interrelated concepts within MH. MH is therefore a state of complete well-being whereby individuals can realize their inherent potential, live out their strengths, are able to cope with normal everyday stressors, and can make a valuable contribution to society (World Health Organization (WHO), 2004, p. 10). In other words, MH is not just about avoiding the conditions that attribute to misery and suffering but rather an active strive toward finding the conditions that lead to happiness and “the good life” (Seligman, 2012).

According to Keyes (2002), MH problems could affect anyone; however, certain population groups are more vulnerable to onset than others. Research suggests that university students are three times more likely to develop MH complaints than the general populous (Auerbach et al., 2016) and is therefore considered a vulnerable group (Ribeiro et al., 2017). Eisenberg et al. (2013) argued that one third of students experience significant MH issues ranging from depression and anxiety to suicidality. These issues stem from stressors such as demanding coursework, time pressure, poor interpersonal relationships with peers/lecturers (Houghton et al., 2012; Basson and Rothmann, 2019; Mertens et al., 2020), social isolation, peer pressure, and study–life imbalances (Bergin and Pakenham, 2015). Studies have shown that students struggle to cope with these demands and, even under “normal” (non-pandemic) circumstances, do not possess the necessary personal resources or SR to buffer against the effects thereof on their MH (Mokgele and Rothmann, 2014). During pandemics, the MH of students could have adverse effects on physical health, life achievement, personal relationships, and life satisfaction as social isolation, fear of infection, and uncertainty take their toll (Lau et al., 2005; Brooks et al., 2020). These MH problems lead to significant impairment in psychological functioning, which negatively affects academic performance, academic throughput, and learning potential (Ebert et al., 2018; Meeter et al., 2020). Although a significant amount of research has been conducted on the antecedents and outcomes of students’ MH, it is unsure which factors and to what extent these would be applicable during the COVID-19 pandemic (Fried, 2020; Mertens et al., 2020).

### SD and SR of Students

The SD and SR framework (SDRF: Mokgele and Rothmann, 2014) may act as a functional theoretical framework to explain which factors may influence university students’ MH during the COVID-19 lockdown. This model, drawing from the job–demands–resources model (Demerouti et al., 2000), states that students’ MH is a function of a dynamic interaction between study characteristics (SD and SR) and its (de)energizing and motivational consequences (Mokgele and Rothmann, 2014). This model proposes that the fundamental characteristics of students’ educational experiences at university can be classified into either SD or SR.

SD refer to various factors that require sustained physical/psychological effort over time that may result in stress when such exceeds students’ personal limits (Mokgele and Rothmann, 2014). These factors may include study load, time pressures, and educational volatility (Kember, 2004). When students can balance the demands from university with their inner capacity, they may perform better through optimizing their learning experiences. However, when SD exceed the capacity of the individual, it may lead to negative consequences such as burnout, depression, or anxiety (Lesener et al., 2020).

In contrast, SR refer to enabling factors that promote engagement and guard against the onset of MH problems (Mokgele and Rothmann, 2014). When the external environment lacks resources, students cannot reduce the potentially harmful influence of SD on MH, leading to an inability to perform (Kember, 2004) adequately. However, resource availability enhances study engagement, strengthening MH (Cilliers et al., 2018). Several studies have shown that specific SR (i.e., peer support, lecturer support, growth opportunities, and information availability) may enhance students’ MH (Kember, 2004; Mokgele and Rothmann, 2014). Students’ social and academic inclusion into the university environment is fostered through lectures, discussions with peers, campus involvement, and learning communities (Mokgele and Rothmann, 2014). Therefore, social support mechanisms like these are essential SR needed to promote the MH of students.

The dynamic interaction between SD and SR activates either an (de)energizing or motivational process. Schaufeli and Bakker (2004) argued that when there is a disproportional balance between SD, available resources, and the student’s ability to cope, it leads to mental exhaustion and fatigue, which negatively affects MH. In contrast, when students have sufficient study-related resources, it acts as a buffer against the effect of study-related demands on their MH and leads to engagement and study motivation (Mokgele and Rothmann, 2014). When study engagement is high and psychological distress is low, it leads to positive rates of change in students’ overall MH, which affects their academic performance (Schaufeli and Bakker, 2004). In essence, research has shown that SD causally decrease and SR causally increases experiences of MH of university students (Schaufeli et al., 2002; Robins et al., 2015; Lesener et al., 2020).

However, during pandemics, the experiences of SD and SR may be significantly different from those during “normal times,” given the radical changes in both the external environment and the educational modus (Fried et al., 2020). It is therefore not clear how study characteristics may affect students’ MH and to what extent during times of radical change. Therefore, any intervention being implemented by universities aimed at enhancing the MH of students without understanding how SD/SR affects MH may not yield the desired effects (Van Zyl and Stander, 2019).

### Current Study

As such, the present study aimed to investigate the trajectory patterns, rate of change, and longitudinal associations between SR/SD and MH of Dutch students before and during the COVID-19 lockdown. Given the novelty of the situation, no *a priori* hypotheses could be formulated, except that the COVID-19 lockdown procedures adversely affected the trajectories of SR/SD and MH.

## Materials and Methods

### Research Approach and Procedure

A longitudinal survey-based research design was employed to investigate the longitudinal trajectories and associations of SR/SD and MH within a sample of Dutch university students.^1^ Participants were enrolled for a master’s level course on research methodology at a Dutch university. Data collection occurred over a 3 month period during the third quartile of the academic year (January to April 2020) and involved the completion of a battery of electronic psychometric assessments weekly for 7 weeks, with a follow-up assessment 1 month later. Questionnaires were distributed electronically through Qualtrics^TM^. Four weekly self-report assessments were conducted before the COVID-19 lockdown procedures were introduced. A fifth assessment took place during the week of lockdown announcements and university closures, which was followed by two weekly assessments directly after. The final assessment occurred 1 month after the seventh assessment.

Before the first assessment, participants were invited to participate in the study via email. Potential participants were informed of the voluntary nature of the study, and the research procedure was explained. In this email, their rights and responsibilities were highlighted and their confidentiality guaranteed, and they were informed that they could withdraw at any time. Participants were also informed that they may request feedback on their data and that upon request, their individual responses would be removed. Further, to ensure anonymity, participants were requested to create a personal identification code which was to be used to link the different data collection processes to each other after completion. To further ensure anonymity, two research partners not associated with the current university managed the distribution of the questionnaires and ensured that any and all potentially identifiable information of participants were removed from the dataset (e.g., metadata and IP addresses). A separate email account was also set up where participants could direct any questions or queries about the study or discuss any problems they may have encountered throughout the process. All guidelines for Ethical Research Practices by the American Psychological Association as well as local legislation were strictly followed.

In order to ensure quality data, a number of *attention checks* based on the guidelines of Abbey and Meloy (2017) were also implemented in each of the assessment waves. First, two direct queries were mentioned in the instructions of the different subscales of the instruments (e.g., “Please rate item 7 on the scale as Completely Disagree” and “Write the word sky in the textbox and rate it as Absolutely”). Second, the response patterns and completion time were analyzed. A *post hoc* analysis of the response consistency, pattern of responses, and effort was evaluated as a function of the time it took to complete the assessments (Buchanan and Scofield, 2018). When a participant did not pass the attention checks, their response to the given assessment was removed. In total, 33 records were removed.

Finally, data were captured and stored on a secured (SSL encrypted) server in compliance with the research institution’s data management policy and the General Data Protection Regulation (No. 679 of 2016: Articles 7, 13–22).

### Participants and Procedure

A census-based sampling strategy was employed to gather data from 174 master students studying at a university in the Netherlands. Data were screened for response quality (Buchanan and Scofield, 2018), which led to the removal of 33 records from the final data set.

The final sample consisted of 141 participants (*cf*. Table 1). The majority of the participants were male (68.1%), Dutch-speaking (94.3%), Dutch national (94.3%) master students between the ages of 22 and 25 years old (95%).

**TABLE 1:** Characteristics of participants (*n* = 141).

**Item**	**Category**	**Frequency (*f)***	**Percentage (%)**
Gender	Male	96	68.1
	Female	44	31.2
	Other	1	0.7
Age (years)	22–25 years	134	95.0
	26–30 years	7	4.9
Nationality	Dutch	133	94.3
	Other	8	5.67
Home Language	Dutch	133	94.3
	Other	8	5.67

### Measures

In each wave, the mean scores of the following instruments were used as indicators of overall SR/SD and MH.

The *Study Demands and Resources Scale* (Mokgele and Rothmann, 2014) was used to measure the availability of SD and SR. The scale consists of 23 items measuring four specific study resources, namely, *peer support* (e.g., “When necessary, can you ask fellow students for help?”), *lecturer support* (e.g., “Can you discuss study problems with your lecturers?”), *growth opportunities* (“Do your studies offer opportunities for personal growth/development?”), and *information availability* (“Are you kept adequately up-to-date about issues within the course?”) as well as overall SD (“Do you have too much work to do?”). Participants were requested to reflect upon the preceding week and rate items on a 5-point Likert scale ranging from 1 (“Never”) to 5 (“Always”). The scale showed to be a reliable instrument across all eight time points in this study with Cronbach’s alpha ranging from 0.84 to 0.96.

The *Mental Health Continuum Short-Form* (Keyes, 2005) was used to measure overall MH. It consists of 14 self-report items, rated on a 6-point Likert scale ranging from 1 (“Never”) to 6 (“Every Day”), that measured emotional, psychological, and social well-being. Participants were requested to reflect upon the preceding week and indicate to what extent they experienced emotional well-being (e.g., “happy”), social well-being (e.g., “that the way in which our society functions, makes sense to you”), and psychological well-being (e.g., “confident to think or express your own ideas and opinions”). The scale was shown to be a reliable instrument across all eight time points in this study with Cronbach’s alpha ranging from 0.89 to 0.93.

### Data Analysis

Data were processed with SPSS v.26 (IBM Corporation, 2020) and Mplus v.8.4 (Muthén and Muthén, 1998) with the robust maximum likelihood (MLR) estimator. *First*, data normality and the internal consistencies were assessed through descriptive statistics. Multivariate normality was established if skewness and kurtosis ranged between -2 and + 2 (Field, 2020). Cronbach’s alpha was used to determine the level of internal consistency at the lower bound limit, where an alpha value larger than 0.80 was deemed to be acceptable (Field, 2020). Further, to determine the presence of common method bias (CMB), Harman’s single-factor approach was employed (Tehseen et al., 2017).

*Second*, through structural equation modeling (SEM), a series of unconditional latent growth models (LGMs) were estimated to determine the starting point (intercept) and growth trajectories (slopes) of students’ SR, SD, and MH. Mean scores of SR, SD, and MH were created at each time point based on their respective factor scores. These were used as indicators for the LGM. Separate growth models, representing linear, quadratic, and piecewise growth trajectories, were estimated to determine which fitted the data best. For the piecewise LGM, two separate linear growth factors were estimated representing the slopes *before* (Times 1–4) and after (Times 5–8) COVID-19 lockdown measures were implemented. Time 1 was set as point 0 within the analyses. Time 5 represented the breakpoint/knot and indicated as point 4 in the analyses (Wang and Wang, 2020). The first growth trajectory was constrained to [0, 1, 2, 3, 4, 4, 4, 4] and the second growth trajectory to [0, 0, 0, 0, 0, 1, 2, 6]. Piecewise LGM is particularly useful when wanting to compare the rate of change between two substantial periods of interest (Duncan et al., 2013). Model fit was determined through conventional SEM standards (*cf.* Table 2 adapted from Kline, 2011 and Wang and Wang, 2020), and fit indices were used to compare competing LGMs.

**TABLE 2:** Model fit statistics.

**Fit indices**	**Cutoff criterion**	**Sensitive to *N***	**Penalty for model complexity**
**Absolute fit indices**			
Chi-square (χ^2^)	Lowest comparative value between measurement models Significant (*p* > 0.01)	Yes	Yes
**Approximate fit indices**			
Root-mean-square error of approximation (RMSEA)	<0.08 but >0.01 90% CI range does not include 0	Yes	Yes
Standardized root mean square residual (SRMR)	<0.08 but >0.01	Yes	No
**Incremental fit indices**			
Comparative fit index (CFI)	>0.90 but <0.99	No	Yes
Tucker–Lewis index (TLI)	>0.90 but <0.99	No	Yes
Akaike information criterion (AIC)	Lowest value in comparative measurement models	No	No
Bayes information criterion (BIC)	Lowest value in comparative measurement models	No	No

*Adapted from Kline (2011) and Wang and Wang (2020).*

*Finally*, two separate sequential piecewise multi-process LGMs were employed to simultaneously model the growth processes of SR and MH as well as SD and MH and to determine the effect of the former’s intercept and rate of change on that of the latter. Here, the intercept and two slopes of SR/SD were regressed on those of MH.

## Results

### Descriptive Statistics, Internal Consistencies, and CMB

The descriptive statistics and internal consistencies are summarized in Table 3 and showed that SR and SD were not normally distributed (skewness/kurtosis >2; Field, 2016); however, all instruments showed acceptable levels of internal consistency (α > 0.80).

**TABLE 3:** Descriptive statistics and Cronbach’s alphas (*n* = 141).

	**Factor**	**μ**	**σ**	**SK**	**Rku**	**α**
**Study resources**						
1	Week 1	3.53	0.44	–1.49	4.13	0.84
2	Week 2	3.50	0.46	–0.96	3.69	0.88
3	Week 3	3.49	0.47	–1.11	3.40	0.90
4	Week 4	3.46	0.44	–1.17	4.90	0.85
5	Week 5	3.46	0.48	–1.21	4.71	0.90
6	Week 6	3.45	0.47	–1.11	4.60	0.90
7	Week 7	3.46	0.47	–0.98	4.31	0.91
8	Week 8	3.52	0.48	–1.25	4.66	0.91
**Study demands**						
9	Week 1	2.50	0.54	0.88	4.01	0.84
10	Week 2	2.44	0.58	0.57	2.29	0.88
11	Week 3	2.54	0.54	0.66	2.50	0.90
12	Week 4	2.59	0.57	0.97	2.97	0.89
13	Week 5	2.54	0.56	0.53	2.18	0.90
14	Week 6	2.53	0.57	0.91	2.61	0.90
15	Week 7	2.50	0.55	0.47	2.47	0.91
16	Week 8	2.51	0.53	0.55	2.40	0.91
**Mental health**						
17	Week 1	4.33	0.76	–0.50	–0.18	0.89
18	Week 2	4.36	0.74	–0.50	–0.05	0.90
19	Week 3	4.30	0.75	–0.43	0.25	0.91
20	Week 4	4.28	0.72	–0.15	–0.43	0.91
21	Week 5	4.29	0.75	–0.39	0.20	0.92
22	Week 6	4.29	0.75	–0.13	–0.61	0.92
23	Week 7	4.29	0.79	–0.36	–0.18	0.93
24	Week 8	4.29	0.78	–0.34	–0.19	0.92

*μ, mean; σ, standard deviation; SK, skewness; Rku, kurtosis; α, Cronbach’s alpha.*

To determine the presence of CMB, Harman’s single-factor approach was employed. Here, all the items were entered into an unrotated exploratory factor analyses. The principal component analyses showed that no single component could be extracted from each time point and that the common shared variance at each time point was below 35% (Podsakoff et al., 2003). Resultantly, the presence of CMB may be ruled out.

### Unconditional LGM

Next, intercept-only, linear, quadratic, and piecewise unconditional LGMs were separately estimated for SR, SD, and MH. Table 4 presents the model fit indicators and shows that the piecewise LGM fitted the data best for SR [χ^2^_(27,_ *_*N*_*_= 141)_ = 37.04, *p* = 0.09, Tucker–Lewis index (TLI)/comparative fit index (CFI) = 0.99, root-mean-square error of approximation (RMSEA) = 0.05, standardized root mean square residual (SRMR) = 0.08], SD [χ^2^_(27, *N* = 141)_ = 41.49, *p* = 0.05, TLI = 0.99, CFI = 0.98, RMSEA = 0.06, SRMR = 0.08], and MH [χ^2^_(27,_ *_*N*_*_= 141)_ = 25.96, *p* = 0.52, TLI/CFI = 1.00, RMSEA = 0.00, SRMR = 0.05]. Two phases of development for both factors were identified: Phase 1, which is prior to the lockdown procedures (Times 0–4), and Phase 2, which is during lockdown (Times 5–7). The interior knot was set at Time 5.

**TABLE 4:** Unconditional latent growth model fit statistics, unstandardized means, and variances.

**Model**	**χ ^2^**	***df***	***p* value**	**TLI**	**CFI**	**RMSEA**	**SRMR**	**AIC**	**BIC**	**90% CI RMSEA**		**Model Comparison**	**Δχ ^2^**	**Δ *df***	**Δ CFI**
										**LL**	**UL**				
**Study resources**															
M0. Intercept only	86.02	27	0.00	0.92	0.91	0.13	0.12	283.25	333.38	0.096	0.155	M3 vs. M0	−27.94*	0	0.07
M1. Linear	92.22	31	0.00	0.91	0.92	0.12	0.11	279.40	317.74	0.091	0.147	M2 vs. M1	−57.77*	−4	0.06
M2. Quadratic	37.45	27	0.09	0.98	0.98	0.05	0.08	226.15	276.28	0.000	0.090	M3 vs. M2	−0.41	0	0.01
M3. Piecewise	37.04	27	0.09	0.99	0.99	0.05	0.08	221.92	271.26	0.000	0.089	M3 vs. M1	−84.93*	−4	0.07
**Study demands**															
M4. Intercept only	78.62	27	0.00	0.95	0.94	0.12	0.09	991.78	1041.91	0.087	0.147	M7 vs.M4	−37.13*	0	0.04
M5. Linear	85.14	31	0.00	0.94	0.95	0.11	0.09	990.30	1,028.63	0.083	0.140	M6 vs. M5	−35.84*	−4	0.03
M6. Quadratic	49.30	27	0.01	0.98	0.98	0.08	0.08	962.46	1,012.59	0.041	0.110	M7 vs. M6	−7.81	0	0
M7. Piecewise	41.49	27	0.05	0.99	0.98	0.06	0.08	954.65	1,004.79	0.016	0.097	M7 vs. M5	−43.65*	−4	0.03
**Mental health**															
M8. Intercept only	47.71	27	0.01	0.97	0.97	0.07	0.09	1,411.09	1,461.22	0.037	0.107	M11 vs. M8	−11.96*	0	0.03
M9. Linear	38.31	31	0.17	0.99	0.99	0.04	0.06	1,389.98	1,428.31	0.000	0.079	M10 vs. M9	−10.10*	−4	0.00
M10. Quadratic	27.25	27	0.45	0.99	0.99	0.01	0.05	1,383.35	1,433.48	0.000	0.066	M11 vs. M10	−1.29	0	0.01
M11. Piecewise	25.96	27	0.52	1.00	1.00	0.00	0.05	1,382.16	1,432.29	0.000	0.063	M11 vs. M9	−11.75*	−4	0.01

χ^2^, chi-square; df, degrees of freedom; TLI, Tucker–Lewis index; CFI, comparative fit index; RMSEA, root-mean-square error of approximation; SRMR, standardized root mean square residual; AIC, Akaike information criterion; BIC, Bayes information criterion; LL, lower level; UL, upper level; *statistically significant (p < 0.05); I, intercept; S1, linear slope; S2, quadratic slope or Piece 2 slope; Δχ^2^, Satorra–Bentler scaled chi-square difference test. Underlined values = Best Fitting Model.

Table 5 presents the unstandardized estimates and shows that average starting amount at baseline (i.e., the intercept) was significant for all three factors: SR (*I*_*Sr*_ = 3.529, SE = 0.04, *p* < 0.05), SD (I_*Sd*_ = 2.481, SE = 0.05, *p* < 0.05), and MH (*I*_*Mh*_ = 4.348, SE = 0.06, *p* < 0.05). At the start of the semester, students therefore reported above average levels of SR and MH as well as average levels of SD. The variances for all intercepts were significant, indicating interindividual variability. Individual growth trajectories significantly differed from one another around the estimated mean for both factors.

**TABLE 5:** Unconditional piecewise latent growth model results: unstandardized estimates, means, variances, and *t*-values.

**Factor**	**Study resources**			**Study demands**			**Mental health**		
	**Estimate (SE)**	***t*-value**	***p*-value**	**Estimate (SE)**	***t*-value**	***p*-value**	**Estimate (SE)**	***t*-value**	***p*-value**
**Covariances**									
S1 with I	−0.256* (0.10)	–2.68	0.01	−0.015* (0.01)	–2.69	0.01	−0.014 (0.01)	–1.52	0.13
S2 with I	0.002 (0.00)	1.00	0.32	−0.001 (0.00)	–0.28	0.78	0.005 (0.00)	1.17	0.24
S2 with S1	−0.001 (0.00)	–1.47	0.14	−0.002* (0.00)	–2.59	0.01	0.001 (0.00)	–0.27	0.79
**Means**									
I	3.529* (0.04)	95.58	0.00	2.481* (0.05)	54.87	0.00	4.348* (0.06)	71.74	0.00
S1	−0.022* (0.01)	–3.10	0.00	0.015 (0.01)	1.62	0.10	-0.017 (0.01)	–1.63	0.10
S2	0.012* (0.00)	2.86	0.00	−0.006 (0.01)	–1.03	0.30	0.002 (0.01)	0.42	0.68
**Variances**									
I	0.174* (0.05)	3.80	0.00	0.249* (0.03)	7.27	0.00	0.450* (0.06)	7.00	0.00
S1	0.004* (0.00)	3.52	0.00	0.008* (0.01)	4.84	0.00	0.007* (0.00)	2.94	0.00
S2	0.000 (0.00)	0.68	0.50	0.002* (0.00)	2.28	0.02	0.001 (0.00)	0.42	0.68

*Statistically significant (p < 0.05).

**FIGURE 1:**
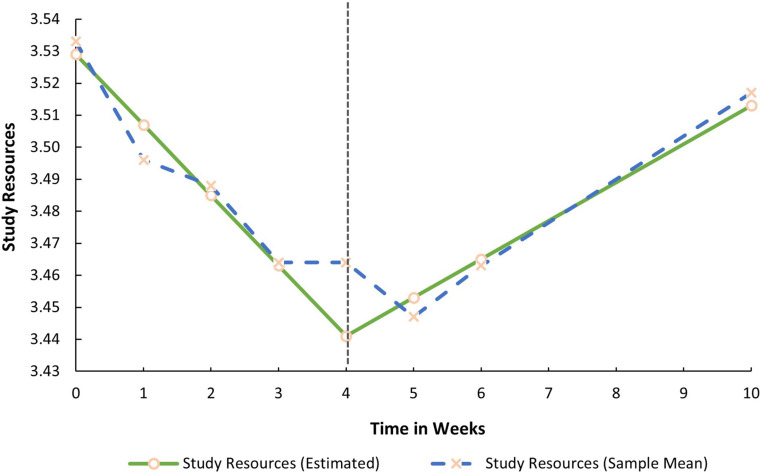
Estimated trajectory of study resources before and during the COVID-19 lockdown.

**FIGURE 2:**
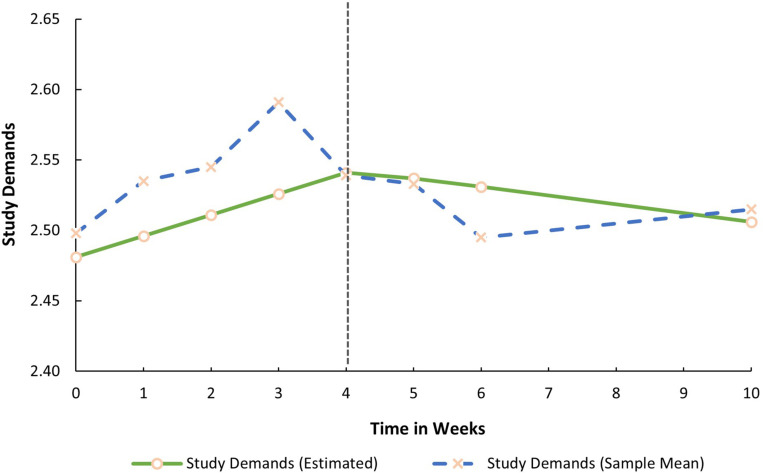
Estimated trajectory of study demands before and during the COVID-19 lockdown.

**FIGURE 3:**
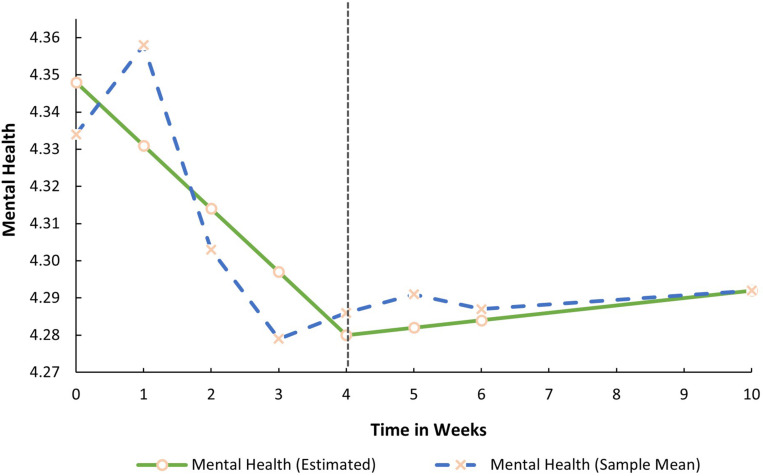
Estimated trajectory of mental health before and during the COVID-19 lockdown.

Both mean growth factors (i.e., the slopes) for SR were significant. This implies that prior to the COVID-19 lockdown procedures, SR decreased in a linear fashion (S1_*Sr*_ = −0.022, *p* < 0.05). During lockdown, it significantly increased by 0.012 base points every week (S2_*Sr*_ = 0.012, *p* < 0.05). In contrast, both latent growth factors for SD (S1_*Sd*_ = 0.015, *p* > 0.05; S2_*Sd*_ = −0.001, *p* > 0.05) and MH were non-significant (S1_*Mh*_ = −0.017, *p* > 0.05; S2_*Mh*_ = 0.002, *p* > 0.05). SD and MH, therefore, remained relatively stable before and during the lockdown. The average trajectories for all three factors are graphically presented in Figures 1–3.

The significant covariance between the intercept and slope 1 of SR [Cov(*I*_*Sr*_, S1_*Sr*_) = −0.256, *p* < 0.05] as well as intercept and Slope 1 of SD [Cov(I_*Sd*_, S1_*Sd*_) = −0.015, *p* < 0.05] indicates that the rate of change in SR/SD was significantly negatively related to its initial levels. Those who reported high levels at baseline had a slightly faster rate of decline prior to the lockdown measures. Further, a significant covariance between Slope 1 and Slope 2 of SD [Cov(S1_*Sd*_, S2_*Sd*_) = −0.002, *p* < 0.05] was found. This implies that the rate of change in SD before the lockdown was negatively associated with the rate of change after the lockdown.

All the remaining covariances between slopes and intercepts in the SR, SD, and MH models were non-significant (*p* > 0.05). During lockdown, levels of SR were therefore not dependent upon the reported mean at baseline, and the rate of change in MH (before and after) was not dependent upon its initial value. Finally, the rate of change in SD during lockdown was not dependent upon its initial levels.

### Sequential Piecewise Multi-Process LGM

Two separate sequential piecewise multi-process LGMs were estimated. First, the association between SR and MH and thereafter the association between SD and MH were estimated.

**FIGURE 4:**
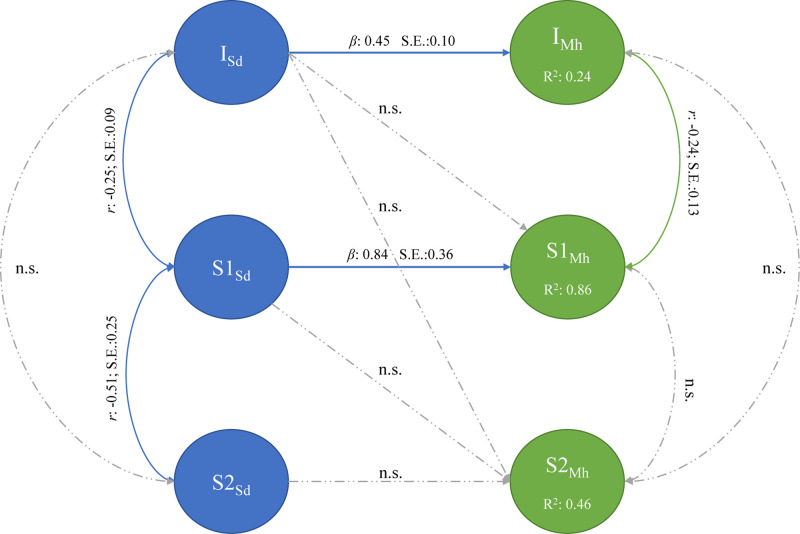
Sequential piecewise multi-process latent growth model: study resources and mental health.

The first sequential piecewise multi-process LGM, presented in Figure 4, aimed to determine the association between the initial state and growth trajectories of SR and MH fitted the data adequately [χ^2^_(113,_ *_*N*_*_= 141)_ = 195.00, TLI/CFI = 0.95, RMSEA = 0.07 (CI:0.054–0.088), SRMR = 0.07]. At baseline, the initial state of SR (*I*_*Sr*_) was positively associated with mean intercept of MH (*I*_*Mh*_) (β = 0.45, SE = 0.10). Higher initial levels of SR therefore influenced initial levels of MH. Further, the rate of change in SR (S1_*Sr*_) prior to the COVID-19 lockdown procedures predicted the rate of change in MH (S1_*Mh*_) in the same phase (β = 0.69, SE = 0.18). This implies that if the rate of change in SR increased before the lockdown procedures, it would positively affect the rate of change in MH.

Further, the covariance between the intercept and pre-lockdown slope of SR indicates that the rate of change in SR is dependent upon its initial position. Higher reported levels in SR is associated with a faster decline before lockdown [Cov(*I*_*Sr*_, S1_*Sr*_) = −0.25, SE = 0.09].

No association between the intercepts and rate of change in SR post lockdown and between the intercepts and slopes of MH could be established. After lockdown, both SR and MH changed at different rates and in different directions. Where MH stays relatively stable before and after lockdown (*I*_*Mh*_ = 4.348, *p* < 0.05, S1_*Mh*_ = −0.017, S2_*Mh*_ = 0.002, *p* > 0.05), SR was reported to increase after lockdown (*I*_*Sr*_ = 3.529, S2_*Sr*_ = 0.012, *p* < 0.05).

**FIGURE 5:**
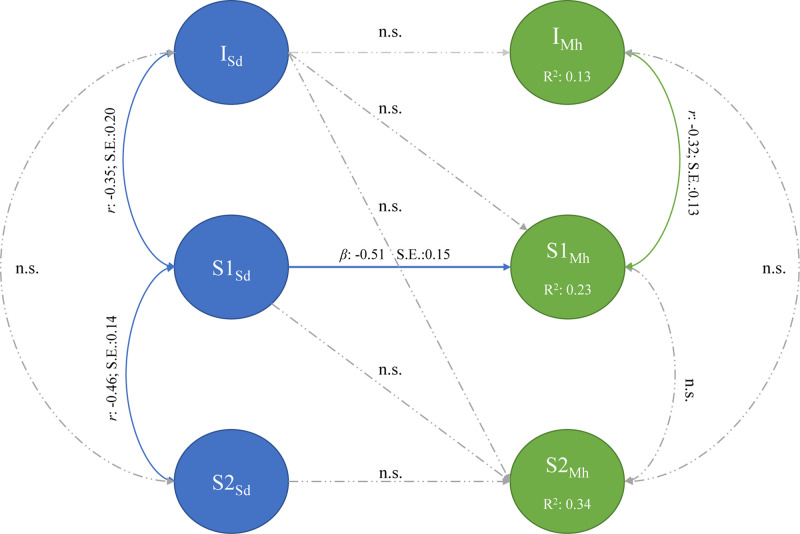
Sequential piecewise multi-process latent growth model: study demands and mental health.

The second sequential piecewise multi-process LGM, presented in Figure 5, aimed to determine whether the association between the initial state and growth trajectories of SD and MH fitted the data adequately [χ^2^_(112,_ *_*N*_*_= 141)_ = 217.75, TLI/CFI = 0.95, RMSEA = 0.08 (CI:0.066–0.098), SRMR = 0.07]. At baseline, the initial state of SD (I_*Sd*_) was not significantly associated with mean intercept of MH (*I*_*Mh*_) (β = −0.14, SE = 0.10; *p* = 0.233). Initial levels of SD therefore did not influence initial levels of MH. Further, the rate of change in SD (S1_*Sd*_) prior to the COVID-19 lockdown procedures was negatively associated with the rate of change in MH (S1_*Mh*_) (β = −0.51, SE = 0.14) during the same phase. This implies that if the rate of change in SD increased before the lockdown procedures, it would negatively affect the rate of change in MH.

The covariance between the intercept and pre-lockdown slope of SD indicates that the rate of change in SD is dependent upon its initial position. Higher reported levels of SD is associated with a faster decline before lockdown [Cov(I_*Sd*_, S1_*Sd*_) = −0.35, SE = 0.10].

No association between the intercepts and rate of change in SD post-lockdown and between the intercepts and slopes of MH could be established. After lockdown, both SD and MH changed at different rates and in different directions. Where MH stayed relatively stable before and after lockdown (I_*Mh*_ = 4.350, *p* < 0.05, S1_*Mh*_ = −0.018, S2_*Mh*_ = 0.003, *p* > 0.05), SD was reported to decrease significantly after lockdown (I_*Sd*_ = 2.480, S2_*Sd*_ = −0.006, *p* < 0.05).

## Discussion

The study employed piecewise LGM to investigate the developmental trajectories and longitudinal association of students’ SR, SD, and MH before and during the COVID-19 lockdown at a Dutch university in the Netherlands. The results showed that moderate levels of SR significantly decreased before, followed by a substantial rate of increase during, the lockdown. In contrast, both SD and MH were reported to be moderate and stable throughout the study. Finally, the growth trajectories of SR and MH were associated only before the lockdown.

### Growth Trajectories in SR, SD, and MH

The results showed that *study-related resources* significantly decreased before and increased during the lockdown. Perceptions of available study-related resources are known to fluctuate throughout a semester/course (Basson and Rothmann, 2019; Mtshweni, 2019). At the beginning of a semester/course, students report an abundance of available SR; however, this starts to dwindle as students progress throughout the semester (Cheng, 2020; Landow, 2006; Mtshweni, 2019). As SD (e.g., workload and time pressure) naturally increase throughout a semester/course, students perceive a decline in the available social study-related resources (peer/lecturer support) needed to effectively cope (Landow, 2006; Waight and Giordano, 2018). Further, given that universities are not adequately staffed to manage high student volumes, educational programs become highly structured with limited lecture contact and access to study-related information (Jack et al., 2018). In turn, this impedes students’ autonomy and distracts from opportunities to grow (Mokgele and Rothmann, 2014). This rate of decline was in line with our findings before the lockdown procedures were announced and implemented. In contrast to our expectations, the findings show that perceptions of available SR increased during the COVID-19 lockdown. This may be due to the university increasing communication frequency relating to how COVID-19 would affect education and how its consequences would be managed (*cf.* Maastricht University, 2020; Technical University of Eindhoven (Tu/e), 2020). The COVID-19 lockdown resulted in courses being taught online, which meant students might have experienced more autonomy in attending to educational activities (e.g., viewing video lectures at their own leisure), having closer contact with lecturers via email or discussion forums, and having the opportunity to negotiate assignment deadlines. Traditionally, university courses are highly structured, and formal communication pertaining to study content and educational matters is within the classroom setting (Landow, 2006). However, given the change to online educational means, the boundaries around and access to lecturers and study-related information were removed.

Further, perceptions of *study-related demands* were relatively stable both before and during the COVID-19 lockdown. No statistically significant changes in these perceptions were reported; however, a slight increase was observed before lockdown and a slight decrease after lockdown. This implies that students did not perceive a difference in study load, time pressure, or study volatility throughout the quartile. This contrasts with the findings of Lesener et al. (2020) who argued that SD increase significantly throughout a given educational period (quartile/semester/course) where the peak is experienced just before the exam. This is a known trend within the tertiary educational environment as students start off within the first week of a course with relatively low SD but need to complete several educational activities (e.g., class tests, assignments, presentations, and interim exams) in short succession of one another (*cf*. Schaufeli et al., 2002; Salanova et al., 2010; Cilliers et al., 2018). This leads to a compounded increase in study-related demands over time (Salanova et al., 2010). As this trend was not fully observed within the current sample, several contextual factors may play an explanatory role. The increased perceptive availability of student-related resources (e.g., information availability, lecturer support, peer support, and growth opportunities) after the lockdown procedures were implemented may have affected the growth trajectories of experienced study-related demands. The SD and SR model argues that SR availability moderates the impact that SD has on negative individual and study-related outcomes (Cilliers et al., 2018; Lesener et al., 2020). In other words, when students’ have sufficient study-related resources, it decreases the potential impact that SD has on their engagement, academic performance, and/or MH (Mokgele and Rothmann, 2014; Cilliers et al., 2018; Lesener et al., 2020).

Finally, the results showed that MH stayed relatively stable throughout the first weeks of the COVID-19 outbreak, which differs from other studies during pandemics. Where others found pandemics like SARS to have precipitated psychological distress (Lau et al., 2005; Brooks et al., 2020), our results show that MH did not significantly change before/during the COVID-19 outbreak. These findings are in line with the findings of both Fried (2020) and Zhang and Ma (2020), who reported that most participants’ MH, in their respective studies, was not negatively affected during the COVID-19 outbreak. Students may be spending more time resting/relaxing and exercising as well as receiving more social support from friends/families (Zhang and Ma, 2020). Fried (2020) also reported that during the first 2 weeks of the COVID-19 lockdown in the Netherlands, students’ level, frequency, and quality of social engagements did not change. Further, students may also start to take more care of their MH needs and have more time to engage in relaxing leisure activities or pursuing new hobbies (Lades et al., 2020). These positive aspects may have offset the potential negative impact of the lockdown on students’ MH (Ebrahim et al., 2020).

### Association Between Trajectories of SR and MH

Within this sample, the growth trajectories of study-related resources and MH were shown to be associated only before lockdown procedures. The average level of study-related resources at baseline was significantly related to the initial state of MH. When students perceive that they have adequate levels of study-related resources at the beginning of the course, it may positively affect the initial levels of their MH (Cilliers et al., 2018). Similarly, the rate of change in both outcomes was positively related before the lockdown procedures. The growth trajectory of study-related resources before lockdown could, therefore, lead to a faster rate of change in MH. This is in line with the tenet of the SD and SR framework (Lesener et al., 2020). Under normal conditions, elevated levels of SR activate a motivational process that increases engagement with study-related content (Lesener et al., 2020). This in turn leads to an increase in MH (Salanova et al., 2010; Basson and Rothmann, 2019). However, during lockdown, study-related resources and MH developed independently and at their own pace.

Further, only the growth trajectories of SD were associated with those of MH before lockdown procedures were implemented. No other direct associations could be established. This implies that when SD increased before the lockdown, it negatively affected MH changes (Lesener et al., 2020). However, given the significant changes in the external environment, both SD and MH stayed relatively the same throughout the assessment period. Therefore, it would seem as though the contextual changes brought on by the COVID-19 lockdown and its impact on the educational system altered the way students perceived the associations between SD, SR, and MH.

### Study Limitations

Despite the novelty of this study, it does present with several limitations. First, given that the COVID-19 lockdown was unexpected and occurred during the study, no pandemic-specific explanatory mechanisms which could have affected the growth trajectories could be measured/controlled. However, the study shows how perceptions of SD, SR, and MH changed from before to during the COVID-19 lockdown. Second, participants in this study originated from a single cohort of students from a university in the Netherlands that comprised mainly males. This limits the generalizability of the findings outside of the given context. Given that the assessments of this study occurred before and during the first COVID-19 lockdown, it would not be possible to collect additional data from other institutions to enhance generalizability. The results may look different for academic institutions in under-resourced institutions. Third, the direction of the effects from SR to MH is based on literature, and analyzing the reciprocal relationships was beyond the scope of this paper. Finally, the paper focused on modeling group-level changes and did not include specific inter-individual differences.

### Recommendations for Future Research

The findings, design, and context of this study also provide a foundation for future research. First, it would be valuable to determine students’ experiences of the lockdown and its impact on MH through focus groups or individual interviews. Second, it is suggested that the “during the pandemic” component of the study be replicated in other European countries and that such be contrasted with more developing countries. Third, an evaluation of the usefulness or effectiveness of newly introduced teaching methods and approaches during the pandemic would be valuable. Finally, it is suggested that future research focus on developing and evaluating educational interventions to enhance students’ MH during the pandemic.

## Conclusion

Despite growing concerns relating to the negative psychological impact of COVID-19 on students, our study shows that no changes in MH were reported during the first lockdown. The university’s measures to manage the impact of the lockdown procedures increased perceptions as to the available study-related resources; but none of the measures or online education processes increased demands. Our study, albeit limited in scope, shows that society’s concern as to the adverse effects of COVID-19 on MH of students may be ill founded in some situations.

## Data Availability Statement

The raw data supporting the conclusions of this article will be made available by the authors, without undue reservation.

## Ethics Statement

Ethical review and approval was not required for the study on human participants in accordance with the local legislation and institutional requirements. The patients/participants provided their written informed consent to participate in this study. All ethical guidelines proposed by the APA and the Helsinki Convention were strictly adhered to.

## Author Contributions

All authors listed have made a substantial, direct and intellectual contribution to the work, and approved it for publication.

## Conflict of Interest

The authors declare that the research was conducted in the absence of any commercial or financial relationships that could be construed as a potential conflict of interest. The reviewer ES declared a shared affiliation with several of the authors, LZ, SR, to the handling Editor at time of review.

## Footnotes

1 The original study was planned to investigate the longitudinal trajectories of the conditions and utility of mental health and academic performance of students throughout the quartile. The aim was to determine the effectiveness of a new study programme and the associated impact thereof on their mental health.

## References

[B1] Abbey J. D. Meloy M. G. (2017). Attention by design: using attention checks to detect inattentive respondents and improve data quality. *J. Oper. Manag*. 53 63–70. 10.1016/j.jom.2017.06.001

[B2] Antonides G. van Leeuwen E. (2020). Covid-19 crisis in the Netherlands: “only together we can control Corona”. *Mind Soc*. 10.1007/s11299-020-00257-x Epub ahead of print.

[B3] Auerbach R. P. Alonso J. Axinn W. G. Cuijpers P. Ebert D. D. Green J. G. (2016). Mental disorders among college students in the World Health Organization World Mental Health Surveys. *Psychol. Med*. 46 2955–2970. 10.1017/S0033291716001665 27484622 PMC5129654

[B4] Basson M. J. Rothmann S. (2019). Pathways to flourishing among pharmacy students: the role of study demands and lecturer support. *J. Psychol. Afr.* 29, 338–345. 10.1080/14330237.2019.1647953

[B5] Bergin A. Pakenham K. (2015). Law student stress: relationships between academic demands, social isolation, career pressure, study/life imbalance and adjustment outcomes in law students. *Psychiatry Psychol. Law* 22 388–406. 10.1080/13218719.2014.960026

[B6] Brooks S. K. Webster R. K. Smith L. E. Wessely S. Greenberg N. Rubin G. J. (2020). The psychological impact of quarantine and how to reduce it: rapid review of the evidence. *Lancet* 395 912–920. 10.1016/S0140-6736(20)30460-8 32112714 PMC7158942

[B7] Buchanan E. M. Scofield J. E. (2018). Methods to detect low quality data and its implication for psychological research. *Behav. Res. Methods* 50 2586–2596. 10.3758/s13428-018-1035-6 29542063

[B8] Cheng C. (2020). Duration matters: peer effects on academic achievement with random assignment in the Chinese context. *J. Chin. Sociol*. 7 1–20. 10.1186/s40711-020-0114-0

[B9] Cilliers J. R. Mostert K. Nel J. A. (2018). Study demands, study resources and the role of personality characteristics in predicting the engagement of first-year university students. *S. Afr. J. High. Educ*. 32 49–70. 10.20853/32-1-1575

[B10] de Haas M. Faber R. Hamersma M. (2020). How COVID-19 and the Dutch ‘intelligent lockdown’change activities, work and travel behaviour: evidence from longitudinal data in the Netherlands. *Transp. Res. Interdiscipl. Perspect*. 6:100150. 10.1016/j.trip.2020.100150 PMC7284275 34171019

[B11] Demerouti E. Bakker A. B. Nachreiner F. Schaufeli W. B. (2000). A model of burnout and life satisfaction amongst nurses. *J. Adv. Nurs.* 32, 454–464. 10.1046/j.1365-2648.2000.01496.x 10964195

[B12] Duncan T. E. Duncan S. C. Strycker L. A. (2013). *An Introduction to Latent Variable Growth Curve Modeling: Concepts, Issues, and Application.* New York, NY: Routledge Academic.

[B13] Dutch Government. (2020). *The Approach to Tackling Coronavirus in the Netherlands.* Available online at: https://www.government.nl/topics/coronavirus-covid-19/tackling-newcoronavirus-in-the-netherlands (accessed April 24, 2020).

[B14] Ebert D. D. Auerbach R. Cuijpers P. Demyttenaere K. Buntrock C. Weisel K. K. (2018). Prediction of major depressive disorder onset in college students. *Depress. Anxiety* 36 294–304. 10.1002/da.22867 30521136 PMC6519292

[B15] Ebrahim S. H. Ahmed Q. A. Gozzer E. Schlagenhauf P. Memish Z. A. (2020). Covid-19 and community mitigation strategies in a pandemic. *BMJ* 368:m1066. 10.1136/bmj.m1066 32184233

[B16] Eisenberg D. Hunt J. Speer N. (2013). Mental health in American colleges and universities: variation across student subgroups and across campuses. *J. Nerv. Ment. Dis*. 201 60–67. 10.1097/nmd.0b013e31827ab077 23274298

[B17] González-Sanguino C. Ausín B. Castellanos M. Á. Saiz J. López-Gómez A. Ugidos C. (2020). Mental health consequences during the initial stage of the 2020 Coronavirus pandemic (COVID-19) in Spain. *Brain Behav. Immun.* 87, 172–176. 10.1016/j.bbi.2020.05.040 32405150 PMC7219372

[B18] Field A. (2016). *An Adventure in Statistics: the Reality Enigma.* New York, NY: Sage Publications.

[B19] Field A. (2020). *Discovering Statistics Using IBM SPSS statistics*, 5th Edn. London. Sage Publications

[B20] Fried E. I. (2020). Mental health and social contact during the COVID-19 pandemic: an ecological momentary assessment study. *PsyArXiv [Preprint].* 10.17605/OSF.IO/MVDPE

[B21] Fried E. I. Papanikolaou F. Epskamp S. (2020). Mental health and social contact during the COVID-19 pandemic: an ecological momentary assessment study. *PsyArXiv* 10.31234/osf.io/36xkp

[B22] Gao J. Zheng P. Jia Y. Chen H. Mao Y. Chen S. (2020). Mental health problems and social media exposure during COVID-19 outbreak. *PLoS One* 15:e0231924. 10.1371/journal.pone.0231924 32298385 PMC7162477

[B23] Greenspoon P. J. Saklofske D. H. (2001). Toward an integration of subjective wellbeing and psychopathology. *Soc. Indic. Res*. 54 81–108.

[B24] Houghton J. D. Wu J. Godwin J. L. Neck C. P. Manz C. C. (2012). Effective stress management: a model of emotional intelligence, self-leadership, and student stress coping. *J. Manag. Educ*. 36 220–238. 10.1177/1052562911430205

[B25] Jack K. Hamshire C. Harris W. E. Langan M. Barrett N. Wibberley C. (2018). “My mentor didn’t speak to me for the first four weeks”: perceived unfairness experienced by nursing students in clinical practice settings. *J. Clin. Nurs*. 27 929–938. 10.1111/jocn.14015 28815761

[B26] IBM Corporation (2020). *IBM SPSS Statistics for Windows, Version 27.0*. Armonk, NY: IBM Corp

[B27] Jacobson N. C. Lekkas D. Price G. Heinz M. V. Song M. O’Malley A. J. (2020). *Flattening the Mental Health Curve: COVID-19 Stay-at-Home Orders Result in Alterations in Mental Health Search Behavior in the United States. PsyArXiv [Preprint].* Available online at: https://psyarxiv.com/24v5b/ (accessed April 24, 2020). 10.2196/19347 PMC7265799 32459186

[B28] Kember D. (2004). Interpreting student workload and the factors which shape students’ perceptions of their workload. *Stud. High. Educ.* 29, 165–184. 10.1080/0307507042000190778

[B29] Keyes C. L. M. (2002). The mental health continuum: from languishing to flourishing in life. *J. Health Soc. Behav*. 43 207–222. 10.2307/3090197 12096700

[B30] Keyes C. L. M. (2005). Mental illness and/or mental health? investigating axioms of the complete state model of health. *J. Consult. Clin. Psychol*. 73 539–548. 10.1037/0022-006X.73.3.539 15982151

[B31] Keyes C. L. M. Yaob J. Hybels C. F. Milstein G. Proeschold-Bell J. R. (2020). Are changes in positive mental health associated with increased likelihood of depression over a two year period? a test of the mental health promotion and protection hypotheses. *J. Affect. Disord*. 270 136–142. 10.1016/j.jad.2020.03.056 32339105

[B32] Kline R. B. (2011). *Principles and Practices of Structural Equation Modelling*, 3rd Edn. New York, NY: Gilford Press.

[B33] Lades L. Laffan K. Daly M. Delaney L. (2020). Daily emotional wellbeing during the COVID-19 pandemic. *PsyArXiv [Preprint].* 10.31234/osf.io/pg6bw PMC7361840 32573074

[B34] Landow M. V. (2006). *Stress and Mental Health of College Students.* Hauppauge, NY: Nova Publishers.

[B35] Lau J. Yang X. Pang E. Tsui H. Y. Wong E. Yun K. W. (2005). SARS-related perceptions in Hong Kong. *Emerg. Infect. Dis*. 11 417–424. 10.3201/eid1103.040675 15757557 PMC3298267

[B36] Leigh-Hunt N. Bagguley D. Bash K. Turner V. Turnbull S. Valtorta N. (2017). An overview of systematic reviews on the public health consequences of social isolation and loneliness. *Public Health* 152 157–171. 10.1016/j.puhe.2017.07.035 28915435

[B37] Lesener T. Pleiss L. S. Gusy B. Wolter C. (2020). The study demands-resources framework: an empirical introduction. *Int. J. Environ. Res. Public Health* 17:5183. 10.3390/ijerph17145183 32709128 PMC7400357

[B38] Maastricht University. (2020). *Information on the Coronavirus (COVID-19).* Available online at https://www.maastrichtuniversity.nl/news/information-coronavirus-covid-19 (accessed October 24, 2020).

[B39] Meeter M. Bele T. Hartogh C. D. Bakker T. de Vries R. E. Plak S. (2020). College students’ motivation and study results after COVID-19 stay-at-home orders. *PsyArXiv [Preprint].* 10.31234/osf.io/kn6v9

[B40] Mertens G. Gerritsen L. Duijndam S. Salemink E. Engelhard I. M. (2020). Fear of the coronavirus (COVID-19): predictors in an online study conducted in march 2020. *J. Anxiety Disord*. 74:102258. 10.1016/j.janxdis.2020.102258 32569905 PMC7286280

[B41] Mokgele K. R. Rothmann S. (2014). A structural model of student wellbeing. *S. Afr. J. Psychol*. 44 514–527. 10.1177/0081246314541589

[B42] Mtshweni V. B. (2019). *The Effects of Sense of Belonging Adjustment on Undergraduate Students’ Intention to Dropout of University.* Master dissertation. University of South Africa, South Africa.

[B43] Muthén L. Muthén B. (1998). *Mplus User’s Guide.* Los Angeles, CA: Muthén & Muthén.

[B44] National Institute for Public Health and the Environment (RIVM) (2020a). *Current Information About COVID-19 (Novel Coronavirus).* Available online at: https://www.rivm.nl/en/novel-coronavirus-covid-19/current-information (accessed November 5, 2020).

[B45] National Institute for Public Health and the Environment (RIVM) (2020b). *Epidemiological Situation of the COVID-19 Situation in the Netherlands: 26 April 2020.* Available online at: https://www.rivm.nl/documenten/epidemiologische-situatie-covid-19-in-nederland-26-april-2020 (accessed April 26, 2020).

[B46] Podsakoff P. M. MacKenzie S. B. Lee J. Y. Podsakoff N. P. (2003). Common method biases in behavioral research: a critical review of the literature and recommended remedies. *J. Appl. Psychol*. 88 879–903. 10.1037/0021-9010.88.5.879 14516251

[B47] Ribeiro ÍJ. S. Pereira R. Freire I. V. Oliveira B. G. D. Casotti C. A. Boery E. (2017). Stress and quality of life among university students: a systematic literature review. *Health Prof. Educ*. 4 70–77. 10.1016/j.hpe.2017.03.002

[B48] Robins T. G. Roberts R. M. Sarris A. (2015). Burnout and engagement in health profession students: the relationships between study demands, study resources and personal resources. *Australas. J. Organ. Psychol*. 8:e1. 10.1017/orp.2014.7

[B49] Roy D. Tripathy S. Kar S. K. Sharma N. Verma S. K. Kaushal V. (2020). Study of knowledge, attitude, anxiety and perceived mental healthcare need in Indian population during COVID-19 pandemic. *Asian J. Psychiatry* 51:102083. 10.1016/j.ajp.2020.102083 32283510 PMC7139237

[B50] Salanova M. Schaufeli W. Martínez I. Bresó E. (2010). How obstacles and facilitators predict academic performance: the mediating role of study burnout and engagement. *Anxiety Stress Coping* 23 53–70. 10.1080/10615800802609965 19326271

[B51] Schaufeli W. B. Bakker A. B. (2004). Job demands, job resources, and their relationship with burnout and engagement: a multi-sample study. *J. Organ. Behav.* 25, 293–315.

[B52] Schaufeli W. B. Martinez I. M. Pinto A. M. Salanova M. Bakker A. B. (2002). Burnout and engagement in university students: a cross-national study. *J. Cross-Cult. Psychol*. 33 464–481. 10.1177/0022022102033005003

[B53] Seligman M. E. (2012). *Flourish: a Visionary New Understanding of Happiness and Wellbeing.* New York, NY: Simon and Schuster.

[B54] Technical University of Eindhoven (Tu/e), (2020). *Information About Coronavirus (COVID-19).* Available online at: https://www.tue.nl/en/our-university/departments/mathematics-and-computer-science/the-department/news/news-overview/01-03-2020-information-about-coronavirus-covid-19/ (accessed November 05, 2020).

[B55] Tehseen S. Ramayah T. Sajilan S. (2017). Testing and controlling for common method variance: a review of available methods. *J. Manag. Sci*. 4 142–168. 10.20547/jms.2014.1704202

[B56] Tull M. T. Edmonds K. A. Scamaldo K. Richmond J. R. Rose J. P. Gratz K. L. (2020). Psychological outcomes associated with stay-at-home orders and the perceived impact of COVID-19 on daily life. *Psychiatry Res*. 289:113098. 10.1016/j.psychres.2020.113098 32434092 PMC7252159

[B57] Van Zyl L. E. Stander M. W. (2019). “Flourishing interventions 2.0: a practical guide to student development,” in *Positive Psychological Intervention Design and Protocols for Multi-cultural Contexts*, eds van Zyl L. E. Rothmann S. (Cham, Switzerland: Springer).

[B58] Waight E. Giordano A. (2018). Doctoral students’ access to non-academic support for mental health. *J. High. Educ. Policy Manag*. 40 390–412. 10.1080/1360080X.2018.1478613

[B59] Wang C. Pan R. Wan X. Tan Y. Xu L. Ho C. S. (2020). Immediate psychological responses and associated factors during the initial stage of the 2019 coronavirus disease (COVID-19) epidemic among the general population in China. *Int. J. Environ. Res. Public Health* 17:1729. 10.3390/ijerph17051729 32155789 PMC7084952

[B60] Wang J. Wang X. (2020). *Structural Equation Modelling: Applications Using Mplus*, 2nd Edn. Chichester: Wiley & Sons.

[B61] World Health Organization (WHO). (2004). Promoting Mental Health : Concepts, Emerging Evidence, Practice: Summary Report / a Report from the World Health Organization, Department of Mental Health and Substance Abuse in Collaboration with the Victorian Health Promotion Foundation and the University of Melbourne. World Health Organization. Available online at: https://apps.who.int/iris/handle/10665/42940 (accessed November 5, 2020).

[B62] World Health Organization (WHO). (2020). *COVID-19 Infection Rates, Deaths and Recovery Dashboard.* Available online at: https://covid19.who.int/ (accessed November 05, 2020).

[B63] Zhang Y. Ma Z. F. (2020). Impact of the COVID-19 pandemic on mental health and quality of life among local residents in Liaoning Province, China: a cross-sectional study. *Int. J. Environ. Res. Public Health* 17:2381. 10.3390/ijerph17072381 32244498 PMC7177660

